# Clinical Remarks on the Cerebral Circulation—Its Pathology

**Published:** 1854-05

**Authors:** Chas. A. Lee


### ART. II.—*Clinical Remarks on the Cerebral Circulation—its Pathology.* By Chas. A. Lee, M. D.

Before proceeding to speak more particularly of the consequences of derangement in the cerebral circulation, I would call your attention to some considerations connected with its peculiarities. That the normal functions of the brain depend on a due and regular supply of arterial blood to the organ, is admitted by all; while there is much diversity of opinion in regard to the pathology of cerebral affections. The opinion was first advanced by Dr. A. Monro, of Edinburgh, in 1783, *that the absolute quantity of blood within the cranium is, at all times, and under all circumstances, nearly the same;* and this opinion has found advocates in Drs. Kelly,* Abercrombie,† Clutterbuck,‡ Watson,‖ Rouchoux,§ Copland,¶ and several other eminent pathologists.

This doctrine is supposed to be sustained by the peculiarities in the cranial structure; the encephalon being included in a strong bony case, which it exactly fills, and removed, it is said, from all atmospheric pressure, except what it receives through the blood-vessels which enter it. Dr. Monro was in the habit of illustrating the doctrine, by filling a glass globe with water, and then inverting it, when no fluid escaped from the small aperture. Dr. Abercrombie states that "it is probable that the quantity of blood circulating in the cerebral vessels cannot be materially increased, except something give way to make room for the additional quantity, because the cavity is already completely full; and it is probable that the quantity cannot be materially diminished, except something entered to supply the space which would

---

* Observations, &c., on the Nervous System. A. Munro, Ed., 1783.

† Path. and Practical Researches on Dis. of Brain and Spinal Cord, &c.

‡ Cyclop. of Prac. Med.; Art. Apoplexy.

‖ Lectures on Prac. of Med., 1st Am. Ed.

§ Researches Sur l'Apoplexia, p. 311. Paris, 1835.

¶ Dic. Prac. Med.; Art. Apoplexy.

become vacant." Dr. Abercrombie seems to have adopted this theory in consequence of the appearances observed in the brains of animals bled to death, in which, while other organs appear blanched and drained of blood, the brains, he says, presents its usual appearance, or even the superficial veins may be found distended with blood. Dr. Clutterbuck, the author of the article on "Cerebral Apoplexy," in the *Cyclopedia of Prac. Medicine*, goes still further, and says, that the amount of blood in the cerebral vessels cannot be diminished by blood-letting or any other means; and that bleeding, of course, can only prove remedial, by diminishing the propulsive power of the heart; and yet, pathologists are constantly speaking of congestions of the blood-vessels of the brain, hyperæmia and anæmia of the organ, determination of blood to the head, cerebral fullness, &c., &c. Dr. Copland, also, one of the ablest pathologists of our times, says, that "the cranial cavity must always contain nearly the same quantity of blood during life, the differences which accrue being chiefly those of rapidity of circulation, and in relative proportion in each part of the series of vessels; an increased quantity in the capillaries, thus causing a disproportionate diminution in the veins." Dr. C., however, in order to explain the pathology of apoplexy, while he assumes that the tissue of the brain is not sensibly compressible, "being lodged in an unyielding bony case," says that the brain may be, nevertheless, "injuriously affected by pressure, chiefly by displacing the contents of its blood-vessels, altering the healthy relative proportion of their contents in each of the series of vessels, and impeding the circulation through a part or whole of the organ;" and it is obvious that pressure on one part must necessarily affect, to a greater or less degree, the whole of the organ, particularly when the pressure is considerable, as by a clot of blood.

The pathologists who adopt these views of cerebral pathology, have to explain the various cerebral affections, as headaches, giddiness, transient derangements of sense, or memory, fits of epilepsy, hysteria, spasms, mania, &c., by variations in the relative quantity of arterial and venous blood, circulating in the brain, or by the variations in the propulsive power of the heart. But neither of these theories, we believe, can satisfactorily explain the phenomena.

If we assume, for example, that the quantity of blood circulating in the brain is always the same, say 20 ounces, and distributed equally between the veins and arteries, as 10 to 10; if the amount in the arteries be, from any cause, increased to 12, then that in the veins must fall to 8, and *vice versa;* the total quantity, according to the hypothesis, remaining always the same. Now it is very evident, as Dr. Mackintosh has suggested, that whether he begin by adding or diminishing, it allows an addition or diminution to the whole quantity of blood in the head; for there can be no increase in the arterial system in the head before a diminution occurs in the veins, nor in the veins before a diminution occurs in the arteries: so that, admitting the theory of invariable quantity to be true, no loss of balance could ever occur in the vessels of the head.

It has also been assumed, without proof, that when the brain is compressed, or pressed on, by a clot of blood, or a depressed portion of bone, that it is the venous circulation of the brain that is chiefly affected, while the arterial is undisturbed, and as the brain, according to the hypothesis, is incompressible, the morbid phenomena must chiefly result from a disturbance in the balance of the arterial and venous circulation. But it is very evident that in such cases, unless the depression or coagulum be directly in the course of the longitudinal or lateral sinuses, every portion of the brain, and all its vessels, must be equally affected. The functions of the brain require, not only that it should receive a large supply of arterial blood, but that the force with which it is sent to the brain should be less, and subject to less variation, than in other parts; which purposes are affected by the tortuosity of the large arteries, and their wide anastomoses, as in forming the circle of Willis; the transit of the large arteries through bone, as the carotid canal of the temporal bone, which prevents any undue distension; also by the arrangement of the vessels in the pria mater, where the arteries divide into innumerable branches and capillaries, freely anastomozing with each other before they enter the brain, into which they pour constant and nearly uniform streams of blood; and then look at the large venous trunks or sinuses, so found as to be scarcely capable of variation in size, either bounded on one side by the cranium, and on the other, by the unyielding tissue of the dura mater, or wholly composed of this thick and tough membrane, so that they are not compressible by any force exerted by the fullness of the arteries, nor capable of distension by an obstruction of the flow of venous blood from the brain. These admirable provisions are well adapted to secure a uniform supply of blood to the brain, and preserve its functions from the frequent disturbances which would otherwise often happen, from all those causes which influence the general circulation.

There is another wise provision which has been overlooked by Abercrombie, Kellie, Copland and other writers on cerebral pathology, which must be taken into the acconnt in all our reasonings and speculations on this subject: and that is, the presence of the cerebro-spinal fluid, which is found beneath the arachnoid, both on the surface of the brain and the spinal cord, and in the cavity of the ventricles. Its office would seem to be, to equalize the bulk of the cranial contents, and to counterbalance the results of differences in the functional cavity of the brain, and in its supply of blood: for it is equally important that the pressure on the walls of the vessels should be regulated, as that there should be a due and regular supply of blood to the organ. The amount of this fluid may vary, from 2, its normal quantity, to 20 ounces, it being increased in cases of atrophy of the brain, and diminished in hypertrophy, or turgescence of its vessels. It acts as a regulator; and Magendie has shown by his experiments, that when it is withdrawn in living animals, it causes great disturbance of the cerebral functions, probably by allowing undue distension of the blood-vessels. But as it is capable of speedy renovation, the nervous centers are soon restored to their natural state. This fluid, then, so rapidly absorbed or generated, according to circumstances, serves not only to equalize the amount of pressure within the cranial but also the spinal cavity: admitting the distension or contraction of the vessels to take place, within certain limits, without any great change in the degree of compression to which the nervous matter is subjected. (Note A.)

The importance of this uniformity of pressure, is evident from a variety of well-known facts. An acquaintance of mine who has lost a portion of the right parietal bone from fracture and subsequent trephining, can at any time be thrown into profound sleep and coma, by gradual pressure over the part, and he wakes up as soon as the pressure is withdrawn. Slow and stertorous breathing, with dilated pupils, &c., are also produced by increasing the pressure, or it may be so graduated as only to cause a tendency to sleep, and sluggishness of the mental faculties, and there can be as little doubt that, in cases of cerebral disturbance connected with general plethora, cardiac hypertrophy, and increased arterial impulse, the symptoms are primarily due to an excess of pressure within the vessels, from an undue quantity of blood, sent into them, just as we find a portion of the cerebral functions resulting from an undue diminution of pressure, as in cases of anemiæ, syncope, &c. The fact is, that the quantity of blood in head varies at different times, as in other organs, though, perhaps, within narrower limits; the softness of the cerebral tissue and its varying functional activity, render it peculiarly liable to undergo alterations in bulk; and Dr. Carpenter states that, in disorderd states of the circulation, the quantity of blood in the vessels of the cranium may be for a time diminished by a sudden extravasation either of blood or serum, into the cerebral substance; and the amount of interior pressure upon the walls of the vessels may also be considerably altered, even when there is no difference in the quantity of fluid contained in them. But these variations only occur in anormal conditions; in health, there can be no doubt that there is a very uniform supply of blood to the brain, owing to the provision, above-mentioned, for guarding against accidental disturbances. The experiments of Dr. Kellie have been chiefly relied on to prove that the brain always contains the same amount of blood, and that it cannot be diminished by bleeding or any other means. The inferences which he deduced from his experiments were,

1st. That a state of bloodlessness is not discovered in the brains of animals which have died by hæmorrhage, but on the contrary, a state of venous congestion.

2d. That the quantity of blood in the cerebral vessels is not affected by gravitation, or posture of the head.

3d. That congestion of the vessels of the brain is not found in those instances where it might be most expected: as in persons who die by hanging, strangulation, suffocation, &c.

4th. That if there be depletion of one set of vessels, arteries or veins, in the cranium, there will be an opposite condition of the other set of vessels.

If we, however, examine carefully these experiments, we find that the above inferences can, by no means, be safely drawn from them. We, on the contrary, find the very opposite conclusions established; for on animals bled to death, the brain contained much less blood than in those destroyed by strangulation or suffocation. This has been clearly established by the experiments of Dr. George Burrows. Dr. B. has proved, most conclusively, that posture has a great influence on vascular congestion of the brain; for in two rabbits, killed by prussic acid, the one suspended by the ears, the other by the legs, while the hearts were still pulsating, he found in the former the membranes and substance of the brain pallid, the sinuses and other vessels being entirely drained of blood; while in the latter, the same parts and vessels were gorged with blood, which, in some places, appeared extravasated. The truth is, that the brain is not exempt from that physical law which causes the gravitation of the fluids to the most depending parts of the body. Dr. Burrows has fully shown that, in the majority of instances, when death takes place by hanging, strangling, suffocation, drowning, and other means of causing apnœa, a congestion of the cerebral vessels is found after death; and that the same condition is also found after death from those diseases which obstruct the return of venous blood from the brain. Where congestion is not present, or strongly marked in such cases, it may be accounted for from the subsidence of the blood, which is facilitated by its fluidity, and the posture of the body after death.

Longet has shown that during inspiration, the circulation of blood in the veins and the return of blood from the head become more rapid, and at the same time the circulation in the arteries becomes slower; and that the reverse of these conditions exists during expiration. Hence it follows that during inspiration the brain contains a diminished quantity of blood, because, in a given time, it receives less arterial and loses more nervous blood; and it follows of necessity that the brain must at one time diminish, and at another increase, either in mass or volume. Longet supposes that the surface of the brain does not descend in inspiration, but that it constantly fills the entire cranial cavity, in consequence of its "rarefaction" or diminished density in inspiration, and its increased density in expiration.

Assuming, however, that the cranium is a bony sphere, within which the brain is completely inclosed, thus removing it from the influence of atmospheric pressure, it would not necessarily follow that no material change can take place in the absolute quantity of blood circulating in the cerebral vessels, as the cerebro-spinal fluid is subject to such great variations. But the experiments of Bourgognon, to prove that the encephalon always exactly fills the cranial cavity, appear by no means conclusive. There are numerous fissures and foramina for the transmission of vessels and nerves through the bones of the cranium, which preclude the idea of its being a perfect sphere, like a glass globe, to which it has been compared. As Dr. Burrows has suggested, if there were not always an equilibrium of pressure on the parts within and without the cranium, very serious consequences would arise at the various foramina of the skull. The vessels entering the cranium are undoubtedly subjected to atmospheric pressure, and this must be transmitted in all directions through the fluid blood, and hence to the encephalon. If, under ordinary circumstances, the brain is exempted from the influence of atmospheric pressure, why are its functions unaffected, when a portion of its walls are removed by trephining or accident. The pressure of the atmosphere upon each square inch of the surface of the body has been estimated at 15lbs.; and the pressure exerted upon the whole body of an adult of ordinary size, is computed at fourteen tons: which is a force more than sufficient to crush him to atoms, were it not opposed by the equal and contrary pressure of the aeriform and other fluids in the interior of the body. Doubtless the brain is situated precisely as all the other organs of the body are; it is exposed to the same atmospheric pressure, which is resisted, as in other parts, by the counter-pressure from within; else, when the cranium is perforated its functions would cease, as the function of a lung ceases when the thoracic wall is opened; and besides, in infancy, and when the brain is most susceptible of injury, the cranial envelop is deficient, and it is not until several months have elapsed before the fontanellès are perfectly closed.

But apart from all theoretical considerations, which are as likely to mislead as to guide us aright, what facts do observation and experience teach in regard to this question? Why, certainly, that there are as great variations in regard to the state of the blood-vessels of the brain as of any other organ. And often, in apoplexy, asphyxia, &c., do we find the cerebral vessels congested, apparently loaded with blood, while in other cases we find them comparatively empty. Tne substance of the brain is not incompressible, as the English pathologists would have us believe, but elastic, and yielding, and allowing room for the expansion of the vessels by a greater quantity of blood. Dr. Burrows has recorded a fact which bears upon this point, which is, that if a patient die soon after blood has been abstracted from the scalp by cupping, and the head be immediately opened, all the exterior and interior anastomosing blood-vessels of the scalp and investing cerebral membranes will be found highly injected, to a circumference corresponding with that of each glass. And then, what does extensive apoplectic effusion teach us with regard to the compressibility of the brain? Here is a large cavity, for example, filled with coagulated blood: must not the rest of the brain have yielded to form this cavity? If so, then the quantity of blood in the brain may vary, for the brain is compressible; unless it is assumed that this space has been gained by emptying the blood-vessels of the brain. But this is a bare assumption, a mere begging of the question, and is not supported by what we observe on dissection, for we find the cerebral vessels generally gorged with blood. If we bleed copiously from the arm, the brain soon feels the influence of the loss of blood; there is giddiness, dimness of vision, ringing in the ears, and, perhaps, convulsions or syncope, which can only be accounted for satisfactorily, by supposing the brain does not receive its usual supply of blood, and then to what measures do we resort for obviating these symptoms? Such, doubtless, as are best calculated to restore the circulation, and favor the usual flow of blood to the head: as, a recumbent position, internal stimulants, &c. But why should posture relieve in syncope, &c., if the brain always contains the same amount of blood? Why, in severe headache, is the pain increased by stooping, or the horizontal posture? In inflammatory affections of the membranes or substance of the brain, depletion from the jugular veins ought to increase the vascular and inflammatory action, inasmuch as the arterial circulation in the brain will be increased in the same ratio — the arteries receiving sufficient to supply what is taken from the veins. And on the same hypothesis, as Dr. Mackintosh has suggested, the converse must also hold good, viz: that when there is great accumulation of blood in the veins of the head, acute action ought to be an impossibility; and the most effectual method of extinguishing inflammation in the brain would be, to place ligatures on the jugulars, or by some other means to prevent the return of blood from the head. If this doctrine were true, then cups, leeches, ice to the head, &c., in inflammatory affections of the brain, should prove injurious rather than beneficial; and the head should be placed in a depending, rather than an elevated position.

There can be no doubt that pressure, within the cranium, exerted through the medium of the blood-vessels, is of great importance, as connected with the functions of the brain, and this pressure is modified by numerous causes. To say that it is always the same, as some writers do, on the assumption that the cerebral substance is chiefly composed of inelastic fluids which are incompressible; or, that "the brain is incompressible by any force exerted through the carotid and vertebral arteries,"* is taking for granted the very thing to be proved. As Dr. Burrows has remarked, the contents of the cranium may be very elastic, and yet very incompressible, as an ivory ball is elastic but incompressible; but these properties bear no constant ratio to each other, as in the sponge, India rubber, ivory, &c. If the blood is incompressible, the vessels are not, for they dilate in proportion to the momentum of blood impelled into them. This is often seen in parts exposed to view, and it is also manifested in the brain when a portion of the skull has been removed, exposing the dura mater, which rises and falls with every systole and diastole of the heart, proving that the brain must receive an impulse from the shock of the arterial pulse, and especially when the cranial covering is entire. The dura mater is most distended during expiration, when the free return of blood from the head is impeded; though Ecker has attributed these movements of the brain chiefly to the ascent of the cerebro-spinal fluid during expiration. I have often watched this alternate rising and sinking of the cerebrum in a girl ten years of age, who had lost nearly the whole right parietal bone, from fracture, and it was interesting to observe, how, from any cause of excitement, as passion, violent exercise, or crying, the movements of the dura mater would become increased; while in repose or sleep, they were comparatively very slight. So that it is obvious that whatever tends to distend the cerebral vessels, must exert a corresponding degree of pressure on the brain. I have also observed, in spinæ bifida, the spinal tumor swelling and becoming tense during prolonged expiration, or during fits of coughing

* Abercrombie.

and crying; and if we watch the fontanelles in the same subject, we shall find, that as the spinal distension increases, that of the fontanelle diminishes, and *vice versa*. Dr. Ecker has showed that when the spinal theca is exposed in a living animal, there is an alternate rising and sinking of the membrane, corresponding with expiration and inspiration; and he maintains that the cerebro-spinal fluid is in constant motion, ascending or descending, which he explains thus: At the moment of expiration, the vertebral sinuses, which are numerous and ramifying exterior to the theca, are distended with blood. This distension must cause an approximation of the theca toward the spinal cord, and this inward or centripetal movement will create a pressure upon the fluid within the theca. This contained fluid seeks an outlet, which it finds more readily toward the inside of the cranium than elsewhere. The cerebral veins are, indeed, distended at this same period, but the unyielding sinuses within the cranium are not dilated in proportion; the spinal fluid can thus partly escape in this direction; it flows in part into the ventricles, and a part probably beneath the arachnoid on the surface of the encephalon. During inspiration, the vertebral sinuses empty themselves, the fluid returns to the vertebral canal, and again occupies the vacated space; and this flux and reflux, he supposes, contributes to the movements of the brain during respiration, when partially deprived of its various coverings. It is believed that this extra-vascular serum is supplemental to the other contents of the cranium, being removable by pressure or absorption; at one time giving place to an increased quantity of blood in the cranium, at another making up for a deficiency of blood in the vessels of the head — acting not only as supplemental to the varying quantity of blood, but also to the variable quantity of nervous matter in the brain: its quantity being in an inverse proportion to the quantity of this nervous matter. Thus, in hypertrophy of the brain, there is a most remarkable deficiency of serum in the cranium: the brain, its ventricles and membranes, are so devoid of this fluid, that they are almost dry; while, on the contrary, in atrophy of this organ, the ventricles and membranes are distended with fluid.

That motions of the brain corresponding with respiration, take place when the skull is entire, it is impossible, of course, to demonstrate, although there is great reason to believe that such is the case, judging from other organs invested with a serous sac. In favor of this belief, we may mention Burrows, Ecker, Burdoch, Magendie, Heureux, &c., while Muller and Longet believe that as long as the bones of the cranium are perfect, no movements of the brain can take place. If the present views in regard to the supplemental character of the spinal fluid, and the variable volume of the encephalon, be correct, then it will follow, necessarily, that such movements do occur.

These views have an important bearing on the pathology and treatment of cerebral affections. There is no class of diseases that require elucidation so much as those of the brain, for there is none whose pathology is more unsettled, or whose treatment is more empirical. For instance, a practitioner of considerable eminence, Dr. Cartwright, of New Orleans, would regard all apoplectic and paralytic seizures, as due to a want of blood in the brain, and would treat them all, indiscriminately, by stimulants; (*Bost. Med. & Surg. Jour.*, Feb., 1854;) while others, of no less reputation, would regard them all as owing to cerebral plethora, and resort to general and local bleeding, in every case. It is one of the most difficult points in practical medicine, to discriminate in these cases, when the same symptoms result from opposite pathological conditions; and yet, such discrimination must be made, if we expect to treat these cases successfully; and yet, after the most careful scrutiny, and aided by all the experience and knowledge at our command, we shall not unfrequently be at a loss in forming our diagnosis, and the true pathology of the case will only be revealed by the effects of our remedies. Copland and the best pathologists of our day, teach us that the brain is more obnoxious to congestion, retarded or interrupted circulation, and compression from vascular fullness, than any other organ, and blood-letting forms the sheet anchor of their remedial resources; and Andral has shown that hypertrophy of the heart is very apt to be associated with sanguineous cerebral effusions, a fact fully confirmed by the observations of Hope, (who found, in 39 cases of apoplexy, hypertrophy of the heart in 28,) Broussais, Lallemand, Bouillaud, Bricheteau and others. But we are never to forget that the symptoms which go under the name of apoplexy, arise from a variety of pathological states, as extravasation of blood in some portion of the brain, from lesion of its vessels; or congestion from violent cardiac action, or obstructed return of blood from the head; or it may be of an asthenic character, and owing to depression, exhaustion, or abolition of cerebral vital influence, giving rise to extravasation of serum, or blood, or congestion. Apoplectic symptoms, too, arise from any cause producing asphyxia and apnœa; or from great exhaustion of the mental and bodily powers, convulsive affections, and violent mental emotions, concussion or shock, and also in diseases of an adynamic type; and the treatment must, of course, be adapted to these various pathological conditions, always paying strict regard to the habits, age, and constitution of the patient, the predisposing and exciting causes; drawing our indications from the countenance, the pulse, especially the carotids, the temperature of the head, and the state of the abdominal functions, secretions, and discharges. Where a state of exhausted or depressed vital power leads to congestion of the capillaries of the brain, and such is doubtless its usual result, such congestion is to be met by stimulants, and powerful revulsives, aided, perhaps, by local depletion to the temples or back of the ears, or internal nostrils, &c.

Under the ordinary circumstances of health, the brain readily accommodates itself to a temporary increase of blood in its vessels (unless this be excessive, or very sudden) owing to the extra-vascular serum and the anatomical peculiarities already mentioned; but in cases of tumors, or hypertrophy of the brain, as in those described by Andral, and that of Dr. Forry, of N. Y., detailed by me in the N. Y. Jour. of Med., we find great disturbance of the cerebral functions, from any cause which much excites the action of the heart, or impedes the return of blood from the head. In the latter case, epileptic attacks were invariably brought on by such causes as mental excitement, severe exercise, &c., and marked relief was obtained by diminishing the *vis a tergo* by general bleeding. A certain degree of pressure upon the brain, then, is absolutely essential to the discharge of its healthy functions; but if it be in excess or deficiency, then derangement occurs. Dr. Abercrombie has related the case of a gentleman, who labored under a considerable degree of deafness, which was invariably relieved by his assuming the horizontal position; and it is a well-known fact that in many persons there is a greatly increased activity of the cerebral functions when in a reclining posture. Bricheteau relates an instance of a student, whose memory was very treacherous, placing himself in a position, in which the head was the most depending part of the body, in order to study with effect.

It is scarcely necessary to add that in syncope, where the phenomena are always the result of insufficient vascular pressure on the brain, the treatment adopted must be such as is calculated to restore, as speedily as possible, the normal degree of pressure, as stimulants, the horizontal posture, &c. Weakened action of the heart, from any cause, will endanger this remedy; and hence the value of the test laid down by Marshall Hall, bleeding in the erect position, to determine the power of the system to sustain the loss of blood, and its value in any given case. So, also, many weakly persons will become faint, by holding the arms in a vertical position above the head, from the diminished pressure on the brain, occasioned by the additional labor imposed upon the heart, of overcoming the effects of gravity on the blood in the upper extremities. In anæmia, the nervous symptoms may be partly owing to the altered state of the blood, partly to its deficient supply, and partly to the diminished vascular pressure on the substance of the brain; and we cannot determine how large a share of the morbid phenomena is owing to any one of these conditions. It is important, however, that we should not confound syncope with apoplexy (as has been done by Dr. Clutterbuck in the *Cyclopedia of Prac. Med., Art. Apoplexy;*) for though there is, in both, a total temporary abolition of the functions of the brain, yet in the one case it is owing to too great, and in the other to too little blood in the brain: in other words, to too little pressure.

In connection with this subject, it is interesting to note the effects which have resulted from cutting off a supply of blood to the head by tying one or both the primitive carotids. In one of Abernethy's cases of ligation of one of the primitive carotids, the patient died soon after in delirium and convulsions. Also, one of Key's and one of Langenbach's patients, within two days after, from "destruction of the functions of the brain." Paralysis occurred immediately in cases recorded by Mayo, Sisco, Molina, and Zeis. Complete hemiplegia occurred in one case of Magendie's, oue of Cooper's, one of Barauero, one of Vincent's, one of Macaulay's, and one of Velpeau's. M. Rossi tied the right subclavian and primitive carotid, and the patient survived six days, when the left primitive carotid and vertebral were fonnd obliterated; of course the brain had been supplied, in the mean time, by the right vertebral artery only. The symptoms are not detailed. Dr. Mott has tied both carotids in two cases, one after an interval of twelve months, "when no inconvenience was experienced when the circulation through the last carotid was interrupted;" in the other, where both carotids were tied after an interval of fifteen minutes "coma and stupor in the course of a few hours supervened, and he died within forty-eight hours." In a case recorded by Dr. Mussey, of Cincinnati, where he tied both primitive carotids after an interval of twelve days, he states that, "though the patient was paler than before, yet he exhibited no signs of a deficient supply of blood to the brain, but walked down two flights of stairs from the operating room, and rode to his lodgings, nearly half a mile, without inconvenience."—*Am. Jour. Med. Science.* "Occasionally," adds Dr. M., "he has had symptoms of cerebral plethora, indicated by pain or a sense of fullness in the head, and a congestion of the vessels of the conjunctiva, from which important relief, or a speedy cure, has been gained by a single bleeding." Prof. Kuhl, Leipzig, tied both primitive carotids, at an interval of twenty-seven days, which produced "heaviness and throbbing in the head, requiring copious bleeding."— (*Velpeau.*) Dr. Ellis, of Michigan, in 1844, tied both the primitive carotids in a case of gun-shot wound of the neck, at an interval of a few hours only, and without any particular influence on the cerebral functions, so far as recorded; though "pulmonary congestion supervened, which required copious blood-letting." The patient perfectly recovered.—(*N. Y. Jour. of Med*, *vol.* v. *p.* 187.) (*See Velpeau's Op. Surgery*, *Art. Aneurism.*)

In a case reported by Acton Key, where he tied the right common carotid in a woman 61 years of age, death occurred from coma in two hours, and, on dissection, the left carotid was found nearly closed, and the vertebrals very small. Dr. Cheevers has published two other cases, (*Lond. Med. Gaz.* vol. vi., p. 703,) where the individuals perished in the course of twenty-four hours after ligation of both primitive carotids; and he has quoted fourteen cases, to show that more or less disturbance of the cerebral functions, as faintness, giddiness, dizziness, loss of speech, delirium, insensibility, &c., occur after ligature of one of the carotids. M. Longet has also quoted sixty-five cases of a similar kind to prove the same fact, (*Anat. et Phys. du Sys. Nerv.*, p. 196. Paris, 1842.) One fact is particularly worthy of note in regard to these cases, and that is, the very great diversity in the effects on the brain in different individuals: some apparently experiencing but slight inconvenience, even when both carotids have been tied; while in others, the operation has proved speedily fatal, and the same dissimilar and conflicting results happen in experiments on the lower animals, though in them, the carotids contribute less to the supply of blood to the brain, than the same vessels do in the human subject.

[To be continued.]

